# MicroRNA profiling in plasma samples using qPCR arrays: Recommendations for correct analysis and interpretation

**DOI:** 10.1371/journal.pone.0193173

**Published:** 2018-02-23

**Authors:** Andreas B. Gevaert, Isabel Witvrouwen, Christiaan J. Vrints, Hein Heidbuchel, Emeline M. Van Craenenbroeck, Steven J. Van Laere, Amaryllis H. Van Craenenbroeck

**Affiliations:** 1 Laboratory of Cellular and Molecular Cardiology, Research Group Cardiovascular Diseases, Department of Translational Pathophysiological Research, University of Antwerp, Antwerp, Belgium; 2 Department of Cardiology, Antwerp University Hospital (UZA), Edegem, Belgium; 3 Translational Cancer Research Unit, Department of Medical Oncology, University of Antwerp, Antwerp, Belgium; 4 Laboratory of Experimental Medicine and Pediatrics, University of Antwerp, Antwerp, Belgium; 5 Department of Nephrology, Antwerp University Hospital (UZA), Edegem, Belgium; Northwestern University, UNITED STATES

## Abstract

MicroRNA (miRNA) regulate gene expression through posttranscriptional mRNA degradation or suppression of translation. Many (pre)analytical issues remain to be resolved for miRNA screening with TaqMan Low Density Arrays (TLDA) in plasma samples, such as optimal RNA isolation, preamplification and data normalization. We optimized the TLDA protocol using three RNA isolation protocols and preamplification dilutions. By using 100μL elution volume during RNA isolation and adding a preamplification step without dilution, 49% of wells were amplified. Informative target miRNA were defined as having quantification cycle values ≤35 in at least 20% of samples and low technical variability (CV across 2 duplicates of 1 sample <4%). A total of 218 miRNA was considered informative (= 59% of all target miRNA). Different normalization strategies were compared: exogenous Ath-miR-159a, endogenous RNA U6, and three mathematical normalization techniques: geNorm (Qbase, QB) and NormFinder (NF) normalization algorithms, and global mean calculation. To select the best normalization method, technical variability, biological variability, stability, and the extent to which the normalization method reduces data dispersion were calculated. The geNorm normalization algorithm reduced data dispersion to the greatest extent, while endogenous RNA U6 performed worst. In conclusion, for miRNA profiling in plasma samples using TLDA cards we recommend: 1. Implementing a preamplification step in the TLDA protocol *without* diluting the final preamplification product 2. A stepwise approach to exclude non-informative miRNA based on quality control parameters 3. *Against* using snoRNA U6 as normalization method for relative quantification 4. Using the geNorm algorithm as normalization method for relative quantification.

## Introduction

MicroRNA (miRNA) are potent regulators of gene expression at the posttranscriptional level. These short non-coding single-stranded RNA molecules influence the translation of specific messenger RNA targets through (in)complete base pairing, inducing translational repression or degradation of the messenger RNA [1]. One miRNA may influence transcription of up to 200 messenger RNAs and more than 50% of the human protein-coding genes are under miRNA regulatory control [2]. Besides being released during cell death or injury, miRNA are also actively secreted by living cells. Bloodborne miRNA are packed in intracellular exosomes, microparticles, Ago proteins and high density lipoprotein cholesterol which renders them very stable and attractive as disease biomarkers. Moreover, miRNA also play an active role in cellular crosstalk: they are taken up by recipient cells and modulate local protein expression [3,4].

Therefore, miRNA–based therapy could be a novel and promising approach for a wide range of pathologies including cancer and cardiovascular diseases [2,5,6].

Whereas miRNA hold certain diagnostic and therapeutic promises, accurate quantification in plasma remains challenging due to the relatively small amounts, small size, sequence homology with precursor forms and lack of reference miRNA [3,4,7]. Preanalytical considerations and modifications to the qPCR protocol specific to the amplification of circulating miRNA have been developed [8,9]. Subsequently, techniques for high-throughput miRNA amplification emerged that allow for large-scale screening while remaining biologically robust [10]. Good reproducibility and small technical variation in these assays have previously been reported for cell culture and solid tissue samples [11–13].

When it comes to circulating miRNA, especially studied as biomarkers, it appears that data are difficult to validate and are sometimes conflicting between groups, which complicates the direct comparison of studies. The lack of agreement on the preferred normalization strategy, the heterogeneous preanalytical protocols (serum versus plasma, use of preamplification and dilution) and use of different commercially available arrays, all could account for this heterogeneity. Suggestions for qPCR quality control and improved data handling have been described [14,15].

Therefore, several protocols regarding 1. RNA isolation, preamplification and dilution, 2. target miRNA performance and 3. data normalization were compared in the present study. Based on these experiments, we aim to propose recommendations for data normalization and improved miRNA quantification specific to miRNA screening in plasma using TaqMan Low Density Arrays (TLDA).

## Results

### 1. RNA isolation, preamplification and dilution

First, the TaqMan Low Density Array (TLDA) protocol was optimized using three RNA isolation protocols and three preamplification (PA) dilutions (Table 1). Plasma samples from a single venipuncture in one patient were used for this optimization. Total RNA was extracted in 3 different amounts of nuclease-free water (50, 100 or 150μL). The best raw mean quantification cycle (Cq) and global mean Cq values were derived from the 100μL elution, although the number of amplified wells was low (<20% of total wells) in all protocols without preamplification. Thus, a preamplification step was added. Preamplification product was diluted to 1:4, 1:40 and 1:1 (no dilution), using RNA from a single 100μL elution from the same patient. No dilution of the preamplification product resulted in the highest number of amplified wells (188). The optimal RNA elution—preamplification dilution combination was thus 100μL elution volume and no dilution of preamplification product.

**Table 1:** Yield of different protocols used for microRNA quantification.

Protocol		Amplified wells		Cq values ≤35		Mean Cq		Normalization microRNA Cq			
RNA elution (μL)	Preamplification dilution	Number	Percentage	Number	Percentage	Raw	Global	ath-miR-159a	RNU44	RNU48	U6
**50**	**0**	37	9.63	26	6.77	33.85	33.09	33.12	Undet	Undet	34.93
**150**	**0**	73	19.01	48	12.50	33.34	32.09	31.50	Undet	Undet	35.68
**100**	**0**	70	18.23	48	12.50	33.04	31.84	30.90	Undet	Undet	Undet
**100**	**1:40**	156	40.63	141	36.72	31.83	31.43	26.60	Undet	34.25	34.37
**100**	**1:4**	175	45.57	160	41.67	30.93	30.51	25.85	Undet	34.31	31.70
**100**	**1:1**	188	48.96	173	45.05	30.12	29.65	24.58	Undet	34.60	31.10

RNA isolated from one patient was used for all experiments. Cq = quantification cycle, N/A = not available on plate, TLDA = TaqMan Low Density Array, Undet = not amplified on plate, Raw = mean of raw Cq values, Global = mean of global mean Cq values.

### 2. Target miRNA performance

**Fig 1:**
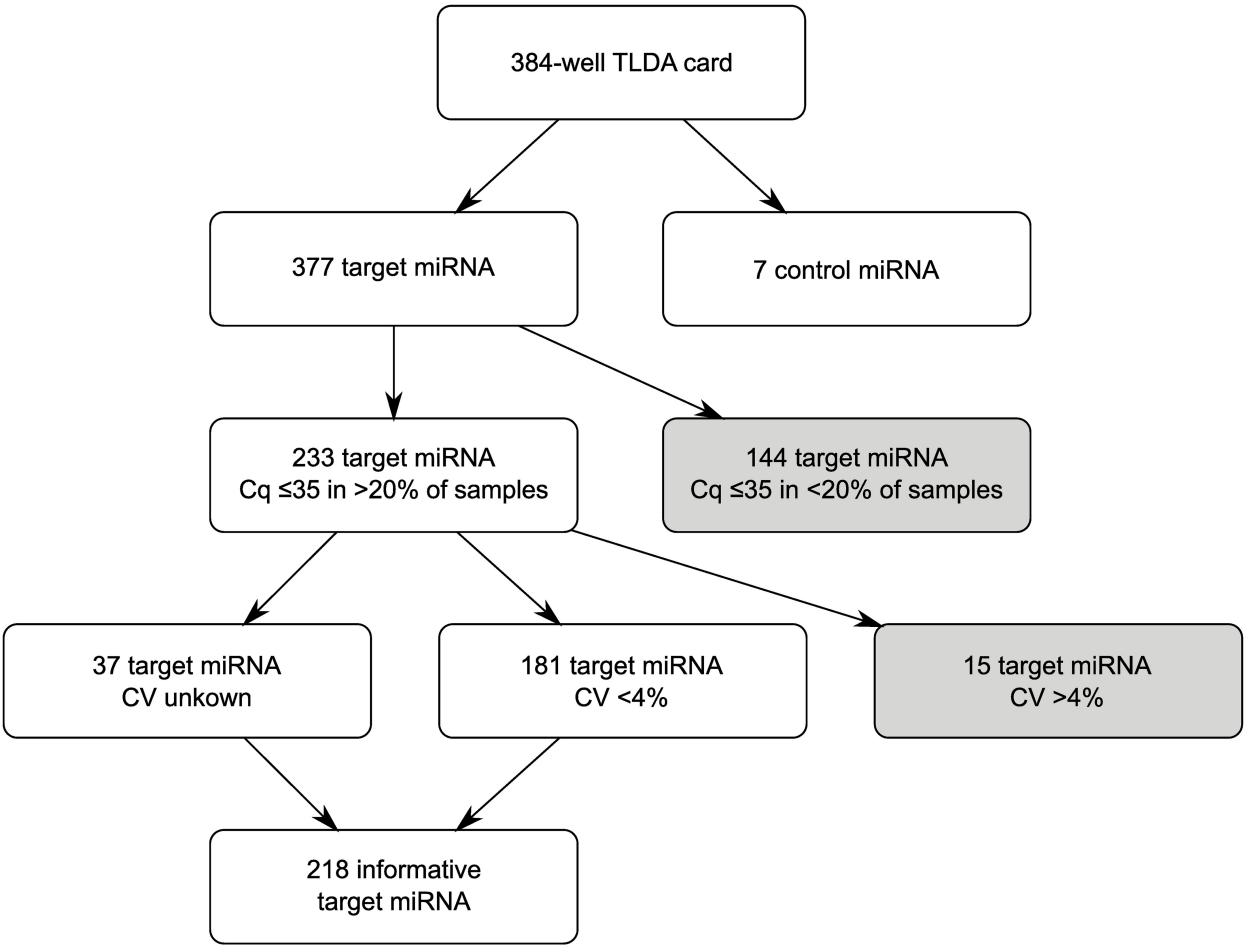
Flowchart displaying selection of informative miRNA targets. From 377 possible target miRNA, 144 are eliminated due to low expression in ≥80% of samples. After exclusion of fifteen target miRNA with a CV ≥4%, 181 target miRNA with a CV<4% are retained (low technical variability). The remaining target miRNA with unknown CV are also retained. The rationale behind the cutoff values of Cq ≤35 and >20% of samples is explained in the Discussion. Cq = quantification cycle, CV = coefficient of variation, miRNA = microRNA, TLDA = TaqMan Low Density Array.

Next, we assessed the performance of the 377 target miRNA present on the TLDA. Data are based on TLDA arrays of plasma samples from 18 clinically stable heart failure patients (mean age 59 years old, 100% male) performed with 100μL elution volume and no dilution of preamplification product. First, low Cq values were assessed for each miRNA. A miRNA was considered non-informative if Cq values were >35 in >80% of samples. Next, coefficient of variation (CV) was calculated for each miRNA in two duplicates of one sample, to assess technical variability. A miRNA was considered non-informative if CV was ≥4%. Of 191 target miRNA in which CV could be calculated, 15 had a CV ≥4%. A flowchart is provided in Fig 1. This strategy resulted in a final selection of 218 informative target miRNA (= 59% of all target miRNA). The miRNA excluded in this two-step algorithm are listed in S1 Table.

### 3. Normalization strategies

As there is no agreement on the ideal normalization strategy for miRNA TLDA arrays, we evaluated five commonly used normalization strategies: *ath-miR-159a* spike-in (Ath), endogenous control RNA (RNU44, RNU48 and U6), global mean (GM), geNorm algorithm (Qbase, QB) and NormFinder algorithm (NF). Details of the normalization methods are provided in Materials & Methods. We evaluated the normalization methods according to their technical variability, biological variability, stability and the extent to which they succeed in reducing data dispersion. RNU44 and RNU48 were not expressed in all samples and are as such not suitable as endogenous controls.

Technical variability was assessed by calculating the CV of the normalization method’s Cq value across 2 duplicates of 1 sample. Technical variability was the lowest for NF (0.04%) and highest for U6 (0.96%). Biological variability was assessed by calculating the CV of the normalization method Cq value across all samples. Biological variability of GM was the lowest (2.13%) and U6 was the highest (9.96%). Stability was assessed by the QB and NF algorithms, resulting in the M and ρ coefficient respectively, with lower coefficients indicating a more stable expression across samples. The M coefficient was lowest in QB (0.0618) and highest in U6 (0.1228). The ρ coefficient was lowest in NF (0.0132) and highest in Ath (0.0598). These results are summarized in Table 2.

**Table 2:** Evaluation of different normalization techniques.

	Median Cq	Technical variability (%)	Biological variability (%)	Stability: M	Stability: ρ
**Ath**	22.37	0.47	4.62	0.1101	0.0598
**GM**	29.52	0.23	2.14	0.0943	0.0550
**NF**	24.02	0.04	7.28	0.0633	0.0132
**QB**	23.93	0.28	7.87	0.0618	0.0197
**U6**	28.96	0.96	9.96	0.1228	0.0436

Technical variability: CV of the normalization method’s Cq value across 2 duplicates of 1 sample. Biological variability: CV of the normalization method’s Cq value across all samples. Ath = *ath-miR-159a* spike-in, CV = coefficient of variation, Cq = quantification cycle, GM = global mean, NF = NormFinder algorithm, M = measure of expression stability used in geNorm algorithm—lower is more stable, QB = geNorm algorithm/Qbase software, ρ = measure of expression stability used in NormFinder algorithm—lower is more stable, U6 = endogenous control RNA U6.

**Fig 2:**
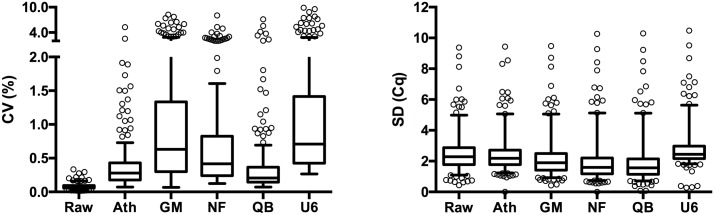
Box and whisker plots of measures of data dispersion: CV (left) and SD (right). First, ΔCq was calculated using the mentioned normalizing method for all 218 miRNA in each sample, then mean ΔCq values and SD were calculated for each miRNA across the 18 samples, finally CV was calculated for each miRNA by dividing SD by mean. Each normalization strategy consistently resulted in an increase in CV compared to the raw data CV. SD decreased in all normalization strategies except for U6 compared to the raw data SD. Ath = ath-miR-159a spike-in normalization, CV = coefficient of variation, Cq = quantification cycle, ΔCq = relative expression of target miRNA (calculated by Cq of target miRNA—Cq of reference gene), GM = global mean normalization NF = NormFinder algorithm normalization, QB = Qbase normalization using geNorm algorithm, Raw = raw (not normalized) data, SD = standard deviation, U6 = U6 endogenous reference RNA normalization.

We also measured the extent to which the normalization methods reduce data dispersion, by calculating CV and SD for each miRNA across all samples. If median CV and median SD are lower for method A than for method B, this means data is more homogenously distributed and biological variability of the target miRNA is reduced by method A. Preferably, the majority of normalized miRNA should have a homogenous distribution with little outliers [16]. SD is reduced most by the QB algorithm (median SD 1.56 vs 2.28 for raw data) while U6 even increased biological variability (median SD 2.44). CV is increased by all normalization methods, but remains lowest using QB (median CV 0.21) and becomes highest using U6 normalization (median CV 0.71). These results are plotted in Fig 2.

**Fig 3:**
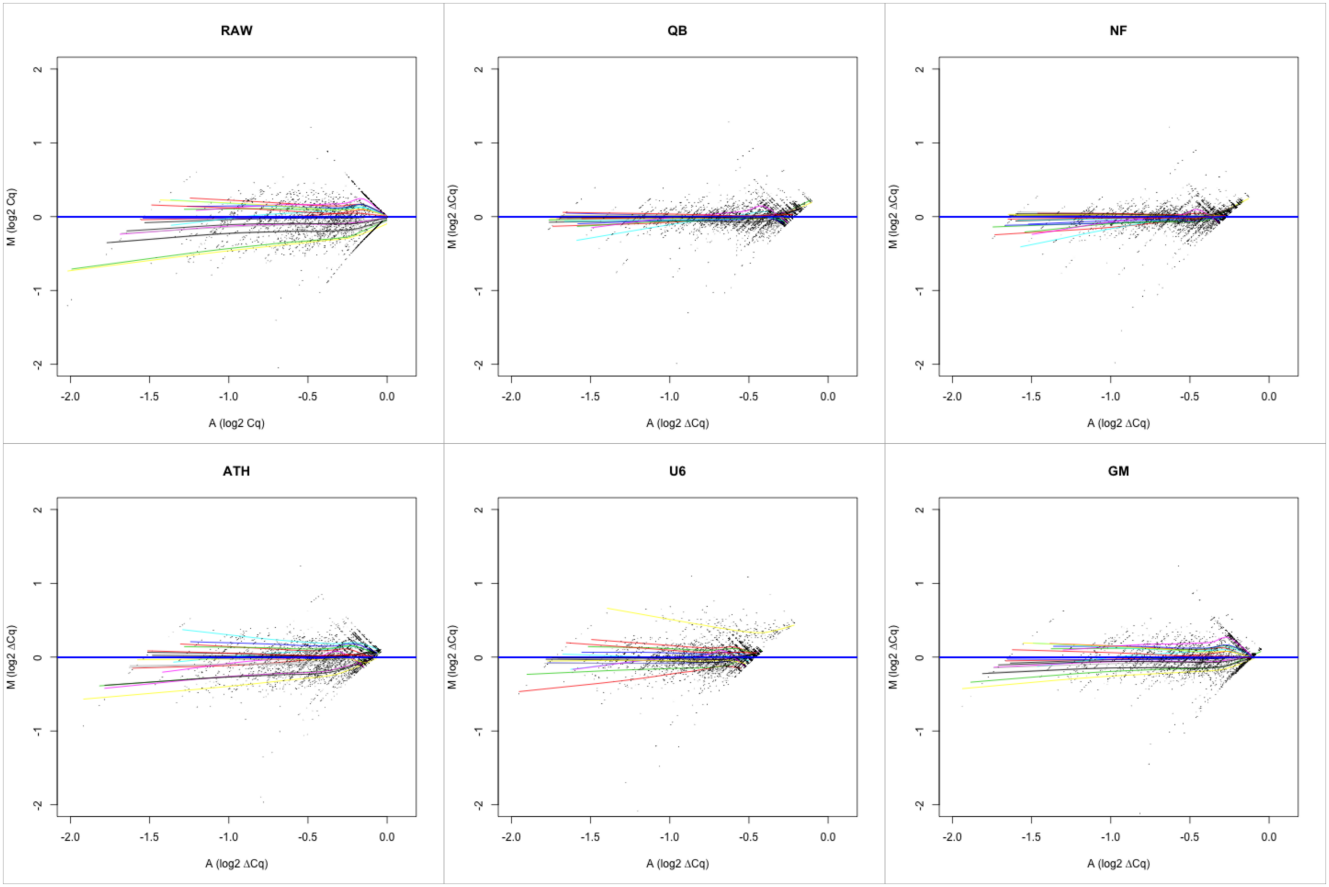
Minus average (MA) plots for each normalization method, with LOESS lines plotted for each sample. Each colored line represents the LOESS line for one sample. NF and QB normalization methods display the straightest LOESS lines closest to zero, i.e. less dispersion compared to Ath, U6 and GM methods and raw data. Ath = ath-miR-159a spike-in, LOESS = local regression, GM = global mean, NF = NormFinder algorithm, QB = geNorm algorithm/Qbase software, U6 = endogenous control RNA U6.

Finally, we drew a minus average (MA) plot. For each miRNA, the difference in Cq value between a sample and the median is plotted against the average of that sample and the median. For each sample, a LOESS (**lo**cal regr**ess**ion) line is plotted using the points from all miRNA. A more linear LOESS line close to zero indicates less data dispersion [17]. In Fig 3, the QB and NF methods display the straightest LOESS lines closest to zero, while U6 and Ath display the widest deviation from zero and least straight LOESS lines.

In conclusion, U6 normalization performed worst on most evaluations, therefore it is not a suitable normalization strategy. QB and NF both performed well on most evaluation criteria, however, QB succeeds better in reducing data dispersion (Fig 2) which is the ultimate goal of data normalization.

### 4. Agreement between different normalization methods

**Fig 4:**
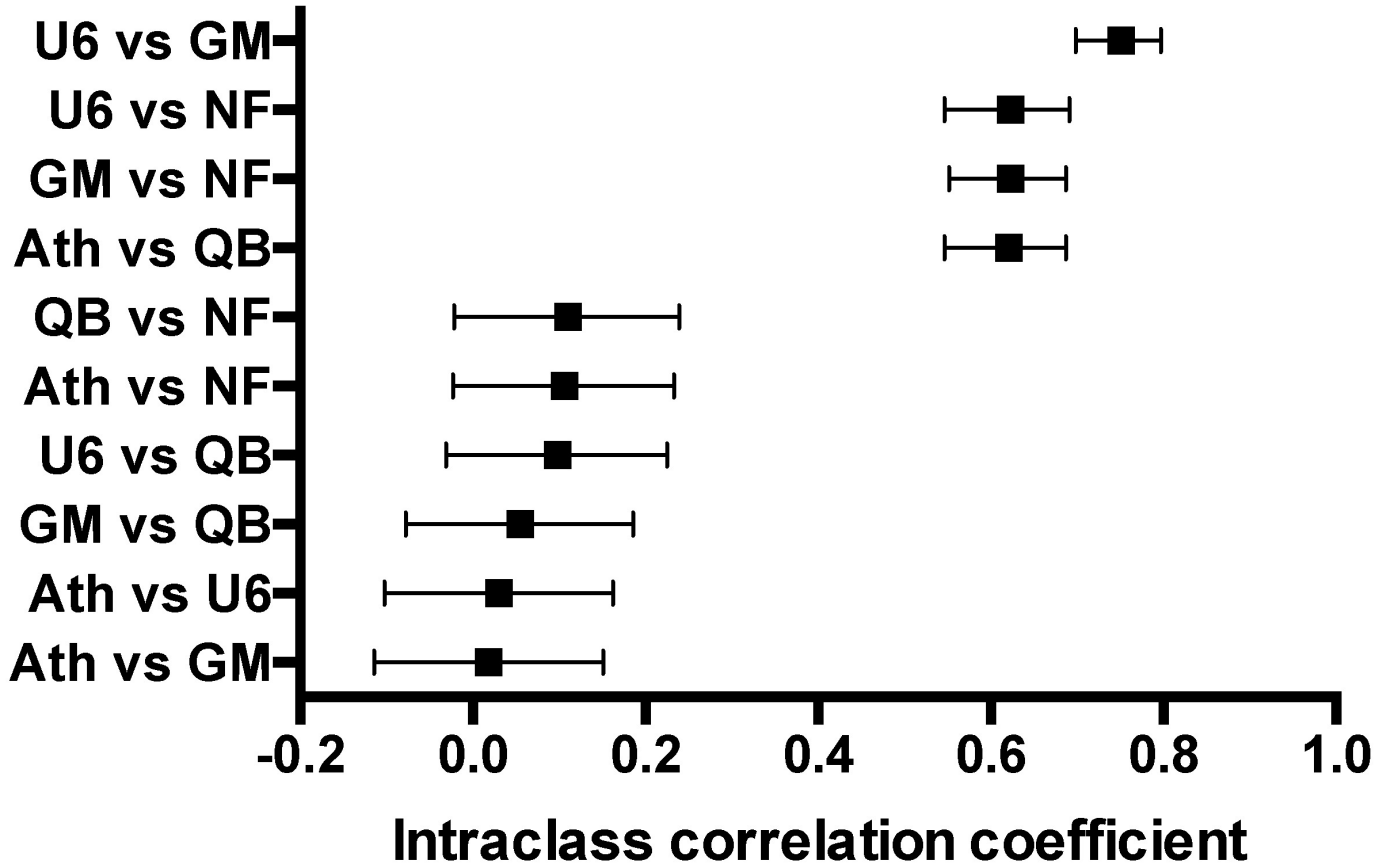
Agreement between different normalization methods. Intra-class correlation coefficient closer to 1 indicates better agreement. Good agreement is seen between U6, GM and NF methods and between Ath and QB methods. Poor agreement is seen between other methods. Ath = ath-miR-159a spike-in, GM = global mean, NF = NormFinder algorithm, QB = geNorm algorithm/Qbase software, U6 = endogenous control RNA U6.

Fig 4 shows agreement between different normalization methods, evaluated using intra-class correlation coefficient. As different normalization methods lead to different ΔCq values in the same sample, the relative expression of a miRNA is influenced by the choice of normalization method. The intra-class correlation coefficient reflects whether method A and method B come to the same results. Striking differences can be appreciated in the agreement between normalization methods, with no method clearly standing out. These results stress the importance of choosing the right normalization strategy.

### 5. Confounders

Multiple linear regression analysis was performed to assess possible confounding factors on the number of amplified wells. Time since RT reaction (p = 0.730), time since RNA isolation (p = 0.184), and time between array loading and qPCR (p = 0.821) were not withheld as confounding factors for the number of amplified wells.

## Discussion

Due to varying preanalytical protocols, different commercially available arrays and the lack of agreement on the preferred normalization strategy, studying circulating miRNA as biomarkers can yield heterogeneous results. For this reason, we addressed technical aspects of large-scale miRNA screening in plasma.

We consider preanalytical (RNA isolation, preamplification) and analytical (miRNA selection, normalization) aspects of miRNA screening in plasma samples using TLDA array cards. We optimize miRNA yield using a specific protocol that can be used in future research, we provide a flowchart for optimal target miRNA selection and we compare commonly used normalization methods.

The number of amplified wells was used as a surrogate for RNA yield. Using plasma samples, a standard TLDA protocol using a 100μL elution resulted in amplification of 70 (18.2%) wells. Changing the RNA elution volume to 50μL lowered the yield, but increasing it to 150μL did not further improve the yield. This indicates that 100μL is sufficient to recover all RNA.

Optimization of the protocol with addition of a preamplification step and no dilution of preamplification product increased the miRNA yield to amplification of 188 (49.0%) wells. This is still lower than previous experiments in solid tissue [18]. This can be explained by the higher relative RNA content of solid tissue versus plasma. While the addition of a preamplification step increases the yield, a major concern is the possibility that the relative miRNA expression levels in the original sample are not maintained [19]. However, using plasma samples, miRNA yield without preamplification is too low for use in profiling experiments. Also, variability introduced by the preamplification step has been proven to be lower than variability introduced by reverse transcription [20,21].

Our final selection of informative miRNA was achieved by implementing a stepwise exclusion based on several quality control parameters. Some aspects of this approach deserve further discussion.

First, Cq values >35 were considered irrelevant. Regarding miRNA qPCR, it is generally accepted that a Cq value of >35 represents unreliably low template detection. Cq values above 35 are, therefore, considered noise [14,19]. It is, however, important to include Cq values >35 AND undetermined Cq values in analysis, as these values contain important biological information (“microRNA is not expressed in this sample”). If one would exclude these values from analysis, this information would be lost. For example, a microRNA that is detected in all samples of group A but not detected in any sample of group B, is importantly differentially regulated between groups A and B. By excluding all missing values, this significant difference would be lost [22]. A better approach is to replace Cq values >35 or undetermined with an arbitrary low value, as we did in our approach [22].

**Fig 5:**
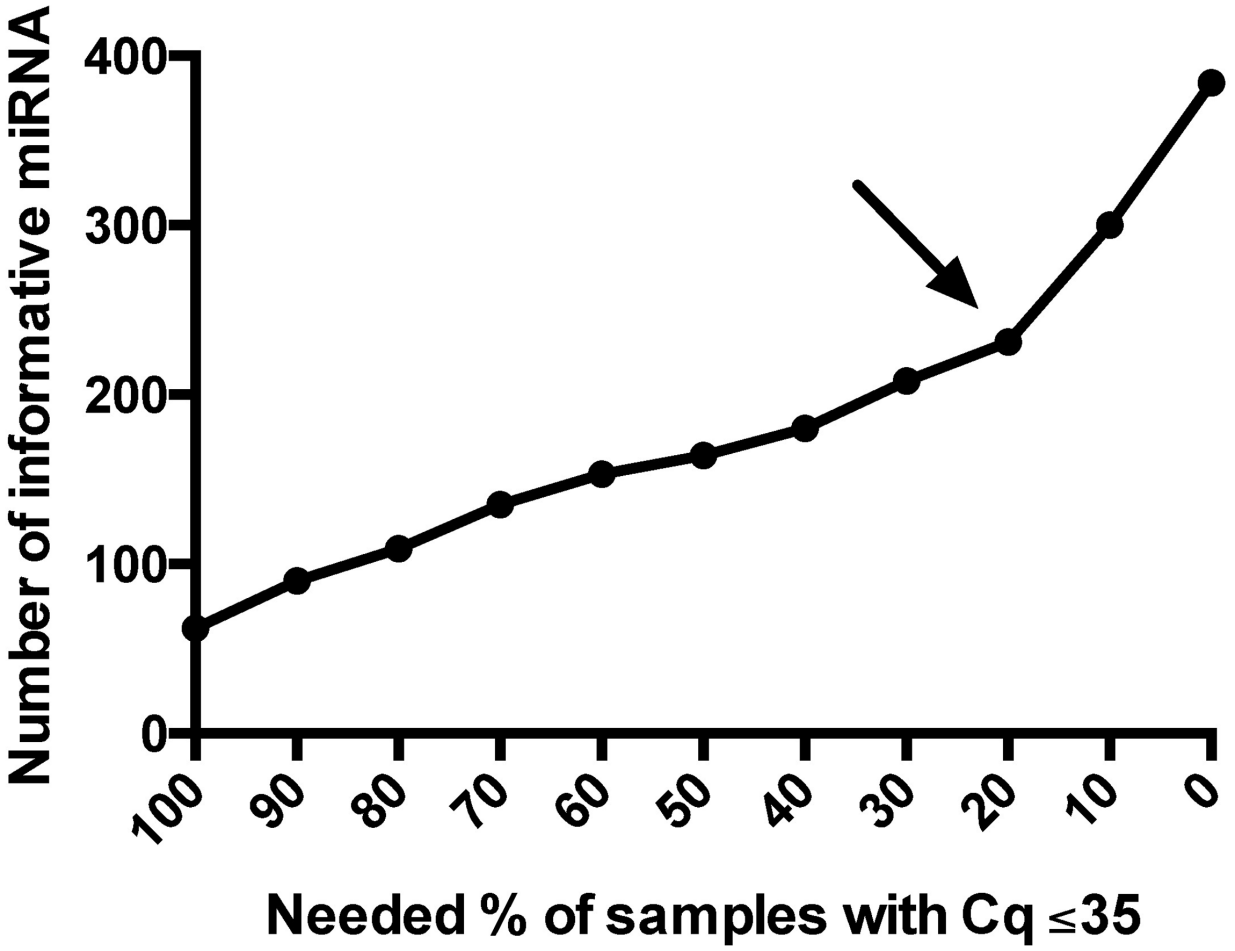
Relationship between number of remaining informative miRNA and the needed percentage of samples with a Cq ≤35. If a miRNA needs to be expressed in all samples (100%) with a Cq ≤35 to be retained, only 62 informative miRNA would remain. If a miRNA does not need to be expressed in any sample (0%) with a Cq ≤35 to be retained, all 377 target miRNA would remain. The elbow of the curve is situated at 20%, which means a miRNA needs to be expressed in 20% of samples with a Cq ≤35. Cq = quantification cycle, miRNA = microRNA.

Second, miRNA with Cq ≤35 in <20% samples were excluded from analysis. These low-expressing miRNA potentially confound statistical analysis by increasing noise. Also, their low expression precludes further use (as biomarker, therapeutic target or both). The cut-off of 20% was chosen based on the observation that the relationship between the accepted percentage and the number of informative miRNA in our experiment is initially linear, but shows a steep increase from 20% onwards (Fig 5).

Third, miRNA with CV values >4% across two duplicates of one sample, indicating high technical variability, were excluded from analysis. The cut-off of 4% is based on previous experiments [23–25]. For 37 target miRNA, technical variability could not be calculated because they were not expressed twice in the duplicate arrays. One could argue to exclude these miRNA from the analysis as possibly their technical variability is high. However, of 233 target miRNA, only 15 (= 6.4%) had high technical variability. When extrapolating this number to the 37 miRNA with unknown technical variability, only 2 or 3 additional miRNA with high technical variability would be detected, at the expense of 34–35 possibly informative miRNA. If the goal of the experiment would be target validation rather than target discovery, a more stringent approach excluding all 37 miRNA with unknown technical variability could be adopted.

One could argue that eliminating non-informative miRNA and Cq values >35 is detrimental to the performance of normalization algorithms, as these algorithms often use noise in their calculation. For completeness, comparison of different normalization methods was repeated with all miRNA and including Cq values >35, and results were not different. We feel it is most correct to use the same dataset for selecting the correct normalization procedure as for all other analyses. As such, the normalization is ideally suited for the data you will actually use.

When using reference genes for normalization, they must have been experimentally validated in specific experimental designs and specific tissues or cell types. Whereas the use of multiple reference genes is generally accepted, the use of a single reference gene is not recommended unless its stability has been proven in identical experimental conditions [26,27]. Despite nearly a decade of experiments, the ideal normalization strategy for TLDA miRNA arrays is still controversial and leads to poor comparison between studies and even paradoxical results. Previously, different normalization methods have been evaluated for TLDA arrays using datasets from solid tissues [16]. The lower RNA yield of plasma samples and the addition of a preamplification step (which possibly introduces additional bias) deserve a separate evaluation. Our experiments show that normalization against endogenous control RNA is not a suitable strategy for plasma samples, which agrees with previous studies [16,28–30]. After normalization the CV value should decrease compared to the raw data [16], as an improved removal of experimentally induced noise implicates a lower CV. However, in our experiment, all normalization strategies led to a general *increase* of the CV compared to the raw data. Deo et al. explained an increase of CV after normalization by the fact that the mean would be close to zero. In that case, the CV would approach infinity and would be sensitive to small changes in the mean. Then, dispersion may be better evaluated using the standard deviation [16]. Many other normalization techniques exist, including LOESS normalization and least-variant set normalization which have both been proposed as superior strategies for array normalization [31,32]. However, these techniques are primarily used to reduce dispersion in raw Cq data, and not for relative quantification. As such, these techniques are less suited for miRNA profiling experiments.

### Conclusion

Currently, a uniform protocol to decrease heterogeneity in studies regarding circulating miRNA is needed. Hence, we make some recommendations for future research in plasma derived circulating miRNA. We propose a minor modification to the existing TLDA protocol to increase the number of amplified miRNA from low-input samples such as plasma. We provide a thorough method to select informative miRNA based on CV and amplification in a minimum of samples. Finally, we evaluate available normalization methods for relative quantification of miRNA.

We translate these results to practical recommendations. For miRNA profiling in plasma samples using TLDA cards:

1. We recommend implementing a preamplification step in the TLDA protocol *without* diluting the final preamplification product.
2. We recommend a stepwise approach to exclude non-informative miRNA based on quality control parameters, outlined in Fig 1.
3. We recommend *against* using endogenous small noncoding RNA as normalization method for relative quantification.
4. We recommend using the geNorm algorithm as normalization method for relative quantification.

## Materials and methods

### 1. Patient population and sample preparation

A total of 19 patients with clinically stable heart failure with reduced ejection fraction were included. After an overnight fast, venous blood was sampled using a 23-gauge needle and collected in tubes containing ethylene diamine tetra acetic acid (EDTA, Vacutainer, BD). The first 3mL of blood was discarded to prevent contamination with skin epithelial cells and endothelial cells. Samples were centrifuged within 30 minutes after collection (1500g, 15min), aliquoted (500μl aliquots) and immediately frozen at -80°C. Median storage time was 2425 days (min. 164 days, max. 3181 days). Median time between isolation and qPCR was 8.5 days (min. 3 days, max. 21 days).

For optimization steps, 3 plasma aliquots from one single venipuncture from one patient were used. From each aliquot, RNA was extracted in a different amount of nuclease-free water (50, 100, 150μL). All actions were performed in an RNase-free environment.

Since 100μL rendered the optimal result, we proceeded with 100μL for the subsequent optimization experiments (preamplification dilution). For assessment of assay quality and differences in normalization, one 500μL aliquot of plasma from each of the remaining 18 patients was used.

In one patient, all experiments were performed in duplicate (starting from the same RNA extract) to assess technical variation.

As the TLDA is a factory-made 384-well card already containing 384 primers, one sample is run on one plate.

Experiments were performed according to the principles expressed in the Declaration of Helsinki. All patients provided written informed consent. The study was approved by the ethical committee of the Antwerp University Hospital (UZA).

### 2. Total RNA extraction in 100μl, 150μl or 50μl nuclease-free water

Aliquoted plasma samples were thawed on ice and centrifuged for 10 min at 4°C (16,000g). Total RNA was isolated from 400μl plasma using a slight modification of the mirVana Paris kit (ThermoFisher, AM1556). As spike-in control 20fmol of Arabidopsis thaliana (ath) miR-159a (ThermoFisher, 4464066) was added to 400μl of plasma and 400μl 2X Denaturing Solution. RNA was extracted using phenol-chloroform and ethanol, centrifuged for 30 minutes at 4°C (16,000g) and washed according to the ‘total RNA’ isolation protocol. To test for differences in RNA yield, total RNA was eluted in 50 μL, 100μl or 150μl preheated nuclease-free water. The aliquoted eluate was stored at -20°C.

### 3. Reverse transcription and qPCR

Reverse transcription and qPCR were performed with MegaPlex primers according to a protocol described previously [18]. Briefly, 3μL total RNA was added to 4.5μl of reverse transcription reaction mix containing TaqMan MicroRNA Reverse Transcription Kit components and MegaPlex Human Pool A RT primers (ThermoFisher, 4399966). 2.5μL reverse transcription product was added to 22.5μL preamplification mix containing MegaPlex Human Pool A PreAmp primers (ThermoFisher, 4399233). Reverse transcription and preamplification were performed on a recently calibrated CFX96 thermal cycler (BioRad, California, USA). Preamplification was performed in all arrays except during optimization of RNA extraction. The preamplification product was diluted with 0.1 x Tris-EDTA in 3 ways for optimization: 1:1 (no dilution), 1:4 (prescribed by the protocol) and 1:40. For subsequent experiments, 1:1 (no dilution) was used. The preamplification product was mixed with TaqMan Universal PCR Master Mix No AmpErase UNG (ThermoFisher, 4324018) and nuclease-free water. TaqMan Low Density MicroRNA Array Human Cards A (ThermoFisher, 4398965) were loaded, sealed and run as prescribed in the protocol. The arrays were run in a recently calibrated 7900HT Fast Real-Time PCR system (ThermoFisher). Thermal cycler conditions are summarized in S2 Table. Primer sequences and miRBase IDs for miRNA on the TLDA card can be found online at https://tools.thermofisher.com/content/sfs/brochures/megaplex-pools-array-card-content.xlsx. Time between reverse transcription and preamplification and qPCR was maximum 72h. Preamplification and qPCR were performed on the same day, with a maximum of 3 arrays per day. Time between array loading and qPCR was maximum 4.5h.

### 4. Normalization strategy

The TLDA contains primers for three small nucleolar RNA proposed as endogenous normalization RNA: RNU44, RNU48 and U6. The TLDA also contains a primer for *ath-miR-159a* (Ath), which can be used as ‘spike-in’ control. In this strategy, a known amount of synthetic Ath is added to the sample before RNA extraction.

We also evaluated three mathematic normalization techniques: geNorm (QB) and NormFinder (NF) normalization algorithms and global mean calculation (GM) [28–30]. QB suggests the geometric mean of *hsa-miR-17* and *hsa-miR-106a* as the most stable normalization. NF proposes to normalize for *hsa-miR-106b*.

### 5. Data analysis

Raw Cq values were calculated in SDS software v.2.4 using automatic baseline and threshold settings. Cq values that were undetermined or >35 were replaced by “Cq = 36” for further analysis to minimize statistical confounding by high quantification cycle values [22]. Relative miRNA levels were expressed as 2^-ΔCq^. Data were analyzed using R version 3.4.3. The geNorm and NormFinder algorithms were obtained from the *NormqPCR* package in R [33]. MA plots were created with the *affy* package in R [34]. Intra-class correlation between two normalization methods was calculated per sample in R using *irr* package. A two-way mixed effects model (average measures) and absolute agreement definition was used. Subsequently, the average of all samples’ ICC + 95%CI between two normalization methods was calculated.

## Supporting information

S1 Table List of microRNA included and excluded in stepwise target selection. Cq = quantification cycle, CV = coefficient of variation.
(DOCX)

S2 Table Thermal cycler conditions for reverse transcription, preamplification and qPCR. qPCR = quantitative polymerase chain reaction.
(DOCX)

S1 Text Raw results of quantitative polymerase chain reaction experiments, expressed as cycle threshold values. (TXT)

S1 Script R code for data cleaning, normalization, minus-average plots and intra-class correlation coefficient calculation. (R)
