# Supplementary material for: MicroRNA profiling in plasma samples using qPCR arrays: Recommendations for correct analysis and interpretation
Source: PLoS One. 2018 Feb 23;13(2):e0193173. doi: 10.1371/journal.pone.0193173 (PMC5825041; doi:10.1371/journal.pone.0193173)
Supplement: S1 Table — Cq = quantification cycle, CV = coefficient of variation. (DOCX) [file pone.0193173.s001.docx]

S1 Table: List of microRNA included and excluded in stepwise target selection.

| **Target** | hsa-let-7a | hsa-let-7c | hsa-let-7d | hsa-let-7e | hsa-let-7f | hsa-let-7g | hsa-miR-1 | hsa-miR-9 | hsa-miR-10a | hsa-miR-10b | U6 snRNA 1 | U6 snRNA 2 | hsa-miR-15a | hsa-miR-15b | hsa-miR-16 | hsa-miR-17 | hsa-miR-18a | hsa-miR-18b | hsa-miR-19a | hsa-miR-19b | hsa-miR-20a | hsa-miR-20b | hsa-miR-21 | hsa-miR-22 | hsa-miR-23a | hsa-miR-23b | hsa-miR-24 | hsa-miR-25 | hsa-miR-26a | hsa-miR-26b | hsa-miR-27a | hsa-miR-27b | hsa-miR-28-3p | hsa-miR-28 | U6 snRNA 3 | U6 snRNA 4 | hsa-miR-29a | hsa-miR-29b | hsa-miR-29c | hsa-miR-30b | hsa-miR-30c | hsa-miR-31 | hsa-miR-32 | hsa-miR-33b |
| --- | --- | --- | --- | --- | --- | --- | --- | --- | --- | --- | --- | --- | --- | --- | --- | --- | --- | --- | --- | --- | --- | --- | --- | --- | --- | --- | --- | --- | --- | --- | --- | --- | --- | --- | --- | --- | --- | --- | --- | --- | --- | --- | --- | --- |
| **Normalization miRNA** |  |  |  |  |  |  |  |  |  |  | X | X |  |  |  |  |  |  |  |  |  |  |  |  |  |  |  |  |  |  |  |  |  |  | X | X |  |  |  |  |  |  |  |  |
| **Excluded based on Cq ≤35 in <20% of samples** |  |  |  |  |  |  |  |  |  |  |  |  |  |  |  |  |  |  |  |  |  |  |  |  |  |  |  |  |  |  |  |  |  |  |  |  |  |  |  |  |  |  |  | X |
| **Excluded based on CV>4%** | X |  |  |  |  |  |  |  |  | X |  |  |  |  |  |  |  | X |  |  |  |  |  |  |  |  |  |  |  |  |  |  |  |  |  |  |  |  |  |  |  |  |  |  |
| **Included** |  | X | X | X | X | X | X | X | X |  |  |  | X | X | X | X | X |  | X | X | X | X | X | X | X | X | X | X | X | X | X | X | X | X |  |  | X | X | X | X | X | X | X |  |

| **Target** | hsa-miR-34a | hsa-miR-34c | hsa-miR-92a | mmu-miR-93 | hsa-miR-95 | mmu-miR-96 | hsa-miR-98 | hsa-miR-99a | hsa-miR-99b | hsa-miR-100 | hsa-miR-101 | hsa-miR-103 | hsa-miR-105 | hsa-miR-106a | RNU44 | hsa-miR-106b | hsa-miR-107 | hsa-miR-122 | mmu-miR-124a | hsa-miR-125a-3p | hsa-miR-125a-5p | hsa-miR-125b | hsa-miR-126 | hsa-miR-127 | hsa-miR-127-5p | hsa-miR-128a | mmu-miR-129-3p | hsa-miR-129 | hsa-miR-130a | hsa-miR-130b | hsa-miR-132 | hsa-miR-133a | hsa-miR-133b | mmu-miR-134 | hsa-miR-135a | hsa-miR-135b | hsa-miR-136 | mmu-miR-137 | hsa-miR-138 | hsa-miR-139-3p | hsa-miR-139-5p | hsa-miR-140-3p | mmu-miR-140 | hsa-miR-141 |
| --- | --- | --- | --- | --- | --- | --- | --- | --- | --- | --- | --- | --- | --- | --- | --- | --- | --- | --- | --- | --- | --- | --- | --- | --- | --- | --- | --- | --- | --- | --- | --- | --- | --- | --- | --- | --- | --- | --- | --- | --- | --- | --- | --- | --- |
| **Normalization miRNA** |  |  |  |  |  |  |  |  |  |  |  |  |  |  | X |  |  |  |  |  |  |  |  |  |  |  |  |  |  |  |  |  |  |  |  |  |  |  |  |  |  |  |  |  |
| **Excluded based on Cq ≤35 in <20% of samples** |  | X |  |  |  | X |  |  |  |  |  |  | X |  |  |  |  |  |  |  |  |  |  |  | X |  | X | X |  |  |  |  |  |  |  | X |  | X |  |  |  |  |  |  |
| **Excluded based on CV>4%** |  |  |  |  |  |  |  | X |  |  |  |  |  |  |  |  |  |  |  |  |  |  |  |  |  |  |  |  |  |  |  |  |  |  |  |  |  |  |  |  |  |  |  |  |
| **Included** | X |  | X | X | X |  | X |  | X | X | X | X |  | X |  | X | X | X | X | X | X | X | X | X |  | X |  |  | X | X | X | X | X | X | X |  | X |  | X | X | X | X | X | X |

| **Target** | hsa-miR-142-3p | hsa-miR-142-5p | hsa-miR-143 | hsa-miR-145 | hsa-miR-146a | hsa-miR-146b-3p | hsa-miR-146b | hsa-miR-147b | hsa-miR-148a | hsa-miR-148b | hsa-miR-149 | hsa-miR-150 | hsa-miR-152 | mmu-miR-153 | hsa-miR-154 | hsa-miR-181a | hsa-miR-181c | hsa-miR-182 | RNU48 | hsa-miR-183 | hsa-miR-184 | hsa-miR-185 | hsa-miR-186 | mmu-miR-187 | hsa-miR-188-3p | hsa-miR-190 | hsa-miR-191 | hsa-miR-192 | hsa-miR-193a-3p | hsa-miR-193a-5p | hsa-miR-193b | hsa-miR-194 | hsa-miR-195 | hsa-miR-196b | hsa-miR-197 | hsa-miR-198 | hsa-miR-199a | hsa-miR-199a-3p | hsa-miR-199b | hsa-miR-200a | hsa-miR-200b | hsa-miR-200c | hsa-miR-202 | hsa-miR-203 |
| --- | --- | --- | --- | --- | --- | --- | --- | --- | --- | --- | --- | --- | --- | --- | --- | --- | --- | --- | --- | --- | --- | --- | --- | --- | --- | --- | --- | --- | --- | --- | --- | --- | --- | --- | --- | --- | --- | --- | --- | --- | --- | --- | --- | --- |
| **Normalization miRNA** |  |  |  |  |  |  |  |  |  |  |  |  |  |  |  |  |  |  | X |  |  |  |  |  |  |  |  |  |  |  |  |  |  |  |  |  |  |  |  |  |  |  |  |  |
| **Excluded based on Cq ≤35 in <20% of samples** |  |  |  |  |  |  |  | X |  |  | X |  |  | X | X |  |  |  |  | X |  |  |  | X | X |  |  |  | X |  |  |  |  |  |  | X |  |  |  |  |  |  |  |  |
| **Excluded based on CV>4%** |  |  |  |  |  |  |  |  |  |  |  |  |  |  |  |  |  |  |  |  |  |  |  |  |  |  |  |  |  |  |  |  |  |  |  |  |  |  |  |  |  |  |  |  |
| **Included** | X | X | X | X | X | X | X |  | X | X |  | X | X |  |  | X | X | X |  |  | X | X | X |  |  | X | X | X |  | X | X | X | X | X | X |  | X | X | X | X | X | X | X | X |

| **Target** | hsa-miR-204 | hsa-miR-205 | hsa-miR-208b | hsa-miR-210 | hsa-miR-214 | hsa-miR-215 | hsa-miR-216a | hsa-miR-216b | hsa-miR-217 | hsa-miR-218 | hsa-miR-219 | hsa-miR-221 | hsa-miR-222 | hsa-miR-223 | hsa-miR-224 | hsa-miR-296-3p | hsa-miR-296 | hsa-miR-299-3p | hsa-miR-299-5p | hsa-miR-301 | hsa-miR-301b | hsa-miR-302a | ath-miR159a | hsa-miR-302b | hsa-miR-302c | hsa-miR-320 | hsa-miR-323-3p | hsa-miR-324-3p | hsa-miR-324-5p | hsa-miR-326 | hsa-miR-328 | hsa-miR-329 | hsa-miR-330 | hsa-miR-330-5p | hsa-miR-331 | hsa-miR-331-5p | hsa-miR-335 | hsa-miR-337-5p | hsa-miR-338-3p | hsa-miR-339-3p | hsa-miR-339-5p | hsa-miR-340 | hsa-miR-155 | hsa-let-7b |
| --- | --- | --- | --- | --- | --- | --- | --- | --- | --- | --- | --- | --- | --- | --- | --- | --- | --- | --- | --- | --- | --- | --- | --- | --- | --- | --- | --- | --- | --- | --- | --- | --- | --- | --- | --- | --- | --- | --- | --- | --- | --- | --- | --- | --- |
| **Normalization miRNA** |  |  |  |  |  |  |  |  |  |  |  |  |  |  |  |  |  |  |  |  |  |  | X |  |  |  |  |  |  |  |  |  |  |  |  |  |  |  |  |  |  |  |  |  |
| **Excluded based on Cq ≤35 in <20% of samples** |  |  | X |  |  |  | X | X | X |  | X |  |  |  |  | X |  | X | X |  |  |  |  | X |  |  |  |  |  |  |  |  |  | X |  |  |  |  | X |  |  |  |  |  |
| **Excluded based on CV>4%** |  | X |  |  | X |  |  |  |  | X |  |  |  |  |  |  |  |  |  |  |  | X |  |  |  |  |  |  |  |  |  |  |  |  |  |  |  |  |  |  |  |  |  |  |
| **Included** | X |  |  | X |  | X |  |  |  |  |  | X | X | X | X |  | X |  |  | X | X |  |  |  | X | X | X | X | X | X | X | X | X |  | X | X | X | X |  | X | X | X | X | X |

| **Target** | hsa-miR-342-3p | hsa-miR-342-5p | hsa-miR-345 | hsa-miR-361 | hsa-miR-362-3p | hsa-miR-362 | hsa-miR-363 | hsa-miR-365 | hsa-miR-367 | hsa-miR-369-3p | hsa-miR-369-5p | hsa-miR-370 | hsa-miR-371-3p | hsa-miR-372 | hsa-miR-373 | hsa-miR-374 | mmu-miR-374-5p | hsa-miR-375 | hsa-miR-376a | hsa-miR-376b | hsa-miR-377 | mmu-miR-379 | hsa-miR-380-3p | hsa-miR-381 | hsa-miR-382 | hsa-miR-383 | hsa-miR-409-5p | hsa-miR-410 | hsa-miR-411 | hsa-miR-422a | hsa-miR-423-5p | hsa-miR-424 | hsa-miR-425-5p | hsa-miR-429 | hsa-miR-431 | hsa-miR-433 | hsa-miR-449 | hsa-miR-449b | hsa-miR-450a | hsa-miR-450b-3p | hsa-miR-450b-5p | mmu-miR-451 | hsa-miR-452 | hsa-miR-453 |
| --- | --- | --- | --- | --- | --- | --- | --- | --- | --- | --- | --- | --- | --- | --- | --- | --- | --- | --- | --- | --- | --- | --- | --- | --- | --- | --- | --- | --- | --- | --- | --- | --- | --- | --- | --- | --- | --- | --- | --- | --- | --- | --- | --- | --- |
| **Normalization miRNA** |  |  |  |  |  |  |  |  |  |  |  |  |  |  |  |  |  |  |  |  |  |  |  |  |  |  |  |  |  |  |  |  |  |  |  |  |  |  |  |  |  |  |  |  |
| **Excluded based on Cq ≤35 in <20% of samples** |  |  |  |  |  |  |  |  |  | X |  |  | X | X | X |  |  |  |  | X | X |  | X | X |  | X |  |  |  |  |  |  |  | X |  |  | X | X | X | X | X |  |  | X |
| **Excluded based on CV>4%** |  |  |  | X |  |  |  |  |  |  |  |  |  |  |  |  |  |  |  |  |  |  |  |  | X |  |  |  |  |  |  |  |  |  |  |  |  |  |  |  |  |  |  |  |
| **Included** | X | X | X |  | X | X | X | X | X |  | X | X |  |  |  | X | X | X | X |  |  | X |  |  |  |  | X | X | X | X | X | X | X |  | X | X |  |  |  |  |  | X | X |  |

| **Target** | hsa-miR-454 | hsa-miR-455-3p | hsa-miR-455 | hsa-miR-483-5p | hsa-miR-484 | hsa-miR-485-3p | hsa-miR-485-5p | hsa-miR-486-3p | hsa-miR-486 | hsa-miR-487a | hsa-miR-487b | hsa-miR-488 | hsa-miR-489 | hsa-miR-490 | hsa-miR-491-3p | mmu-miR-491 | hsa-miR-493 | hsa-miR-494 | mmu-miR-495 | mmu-miR-496 | hsa-miR-499-3p | mmu-miR-499 | hsa-miR-500 | hsa-miR-501-3p | hsa-miR-501 | hsa-miR-502-3p | hsa-miR-502 | hsa-miR-503 | hsa-miR-504 | hsa-miR-505 | hsa-miR-507 | hsa-miR-508 | hsa-miR-508-5p | hsa-miR-509-5p | hsa-miR-510 | hsa-miR-512-3p | hsa-miR-512-5p | hsa-miR-513-5p | hsa-miR-515-3p | hsa-miR-515-5p | hsa-miR-516a-5p | hsa-miR-516b | hsa-miR-517a | hsa-miR-517c |
| --- | --- | --- | --- | --- | --- | --- | --- | --- | --- | --- | --- | --- | --- | --- | --- | --- | --- | --- | --- | --- | --- | --- | --- | --- | --- | --- | --- | --- | --- | --- | --- | --- | --- | --- | --- | --- | --- | --- | --- | --- | --- | --- | --- | --- |
| **Normalization miRNA** |  |  |  |  |  |  |  |  |  |  |  |  |  |  |  |  |  |  |  |  |  |  |  |  |  |  |  |  |  |  |  |  |  |  |  |  |  |  |  |  |  |  |  |  |
| **Excluded based on Cq ≤35 in <20% of samples** |  | X | X |  |  |  | X |  |  |  |  | X | X | X | X |  |  |  |  | X | X | X |  | X | X |  |  | X | X |  | X | X | X |  | X | X | X | X | X | X | X | X | X |  |
| **Excluded based on CV>4%** |  |  |  |  |  |  |  |  |  |  |  |  |  |  |  |  |  |  |  |  |  |  |  |  |  |  |  |  |  |  |  |  |  |  |  |  |  |  |  |  |  |  |  |  |
| **Included** | X |  |  | X | X | X |  | X | X | X | X |  |  |  |  | X | X | X | X |  |  |  | X |  |  | X | X |  |  | X |  |  |  | X |  |  |  |  |  |  |  |  |  | X |

| **Target** | hsa-miR-518a-3p | hsa-miR-518a-5p | hsa-miR-518b | hsa-miR-518c | hsa-miR-518d | hsa-miR-518d-5p | hsa-miR-518e | hsa-miR-518f | hsa-miR-519a | hsa-miR-519d | hsa-miR-519e | hsa-miR-520a | hsa-miR-520a# | hsa-miR-520d-5p | hsa-miR-520g | hsa-miR-521 | hsa-miR-522 | hsa-miR-523 | hsa-miR-524-5p | hsa-miR-525-3p | hsa-miR-525 | hsa-miR-526b | hsa-miR-532-3p | hsa-miR-532 | hsa-miR-539 | hsa-miR-541 | hsa-miR-542-3p | hsa-miR-542-5p | hsa-miR-544 | hsa-miR-545 | hsa-miR-548a | hsa-miR-548a-5p | hsa-miR-548b | hsa-miR-548b-5p | hsa-miR-548c | hsa-miR-548c-5p | hsa-miR-548d | hsa-miR-548d-5p | hsa-miR-551b | hsa-miR-556-3p | hsa-miR-556-5p | hsa-miR-561 | hsa-miR-570 | hsa-miR-574-3p |
| --- | --- | --- | --- | --- | --- | --- | --- | --- | --- | --- | --- | --- | --- | --- | --- | --- | --- | --- | --- | --- | --- | --- | --- | --- | --- | --- | --- | --- | --- | --- | --- | --- | --- | --- | --- | --- | --- | --- | --- | --- | --- | --- | --- | --- |
| **Normalization miRNA** |  |  |  |  |  |  |  |  |  |  |  |  |  |  |  |  |  |  |  |  |  |  |  |  |  |  |  |  |  |  |  |  |  |  |  |  |  |  |  |  |  |  |  |  |
| **Excluded based on Cq ≤35 in <20% of samples** | X | X |  | X |  | X | X |  | X | X | X | X | X |  | X | X | X |  | X | X | X | X |  |  |  | X |  | X | X |  |  | X | X | X |  |  | X | X |  | X | X | X | X |  |
| **Excluded based on CV>4%** |  |  | X |  |  |  |  |  |  |  |  |  |  |  |  |  |  | X |  |  |  |  |  |  |  |  |  |  |  |  |  |  |  |  |  |  |  |  |  |  |  |  |  |  |
| **Included** |  |  |  |  | X |  |  | X |  |  |  |  |  | X |  |  |  |  |  |  |  |  | X | X | X |  | X |  |  | X | X |  |  |  | X | X |  |  | X |  |  |  |  | X |

| **Target** | hsa-miR-576-3p | hsa-miR-576-5p | hsa-miR-579 | hsa-miR-582-3p | hsa-miR-582-5p | hsa-miR-589 | hsa-miR-590-5p | hsa-miR-597 | hsa-miR-598 | mmu-miR-615 | hsa-miR-615-5p | hsa-miR-616 | hsa-miR-618 | hsa-miR-624 | hsa-miR-625 | hsa-miR-627 | hsa-miR-628-5p | hsa-miR-629 | hsa-miR-636 | hsa-miR-642 | hsa-miR-651 | hsa-miR-652 | hsa-miR-653 | hsa-miR-654-3p | hsa-miR-654 | hsa-miR-655 | hsa-miR-660 | hsa-miR-671-3p | hsa-miR-672 | hsa-miR-674 | hsa-miR-708 | hsa-miR-744 | hsa-miR-758 | hsa-miR-871 | hsa-miR-872 | hsa-miR-873 | hsa-miR-874 | hsa-miR-875-3p | hsa-miR-876-3p | hsa-miR-876-5p | hsa-miR-885-3p | hsa-miR-885-5p | hsa-miR-886-3p | hsa-miR-886-5p |
| --- | --- | --- | --- | --- | --- | --- | --- | --- | --- | --- | --- | --- | --- | --- | --- | --- | --- | --- | --- | --- | --- | --- | --- | --- | --- | --- | --- | --- | --- | --- | --- | --- | --- | --- | --- | --- | --- | --- | --- | --- | --- | --- | --- | --- |
| **Normalization miRNA** |  |  |  |  |  |  |  |  |  |  |  |  |  |  |  |  |  |  |  |  |  |  |  |  |  |  |  |  |  |  |  |  |  |  |  |  |  |  |  |  |  |  |  |  |
| **Excluded based on Cq ≤35 in <20% of samples** |  | X |  | X | X | X |  |  |  | X | X | X |  | X |  |  |  |  |  |  | X |  | X |  | X |  |  |  | X | X |  |  |  | X | X | X |  | X | X | X | X |  |  |  |
| **Excluded based on CV>4%** |  |  |  |  |  |  |  |  |  |  |  |  | X |  |  | X |  |  |  |  |  |  |  |  |  |  |  |  |  |  |  |  |  |  |  |  |  |  |  |  |  |  |  |  |
| **Included** | X |  | X |  |  |  | X | X | X |  |  |  |  |  | X |  | X | X | X | X |  | X |  | X |  | X | X | X |  |  | X | X | X |  |  |  | X |  |  |  |  | X | X | X |

| **Target** | hsa-miR-887 | hsa-miR-888 | hsa-miR-889 | hsa-miR-890 | hsa-miR-891a | hsa-miR-891b | hsa-miR-892a | hsa-miR-147 | hsa-miR-208 | hsa-miR-211 | hsa-miR-212 | hsa-miR-219-1-3p | hsa-miR-219-2-3p | hsa-miR-220 | hsa-miR-220b | hsa-miR-220c | hsa-miR-298 | hsa-miR-325 | hsa-miR-346 | hsa-miR-376c | hsa-miR-384 | hsa-miR-412 | hsa-miR-448 | hsa-miR-492 | hsa-miR-506 | hsa-miR-509-3-5p | hsa-miR-511 | hsa-miR-517b | hsa-miR-519c | hsa-miR-520b | hsa-miR-520e | hsa-miR-520f |
| --- | --- | --- | --- | --- | --- | --- | --- | --- | --- | --- | --- | --- | --- | --- | --- | --- | --- | --- | --- | --- | --- | --- | --- | --- | --- | --- | --- | --- | --- | --- | --- | --- |
| **Normalization miRNA** |  |  |  |  |  |  |  |  |  |  |  |  |  |  |  |  |  |  |  |  |  |  |  |  |  |  |  |  |  |  |  |  |
| **Excluded based on Cq ≤35 in <20% of samples** | X | X |  | X | X | X | X | X |  |  |  | X | X | X | X | X | X | X |  |  | X | X | X | X | X | X | X | X | X |  |  | X |
| **Excluded based on CV>4%** |  |  |  |  |  |  |  |  | X |  |  |  |  |  |  |  |  |  |  |  |  |  |  |  |  |  |  |  |  |  |  |  |
| **Included** |  |  | X |  |  |  |  |  |  | X | X |  |  |  |  |  |  |  | X | X |  |  |  |  |  |  |  |  |  | X | X |  |
