# Supplementary material for: MicroRNA profiling in plasma samples using qPCR arrays: Recommendations for correct analysis and interpretation
Source: PLoS One. 2018 Feb 23;13(2):e0193173. doi: 10.1371/journal.pone.0193173 (PMC5825041; doi:10.1371/journal.pone.0193173)
Supplement: S2 Table — qPCR = quantitative polymerase chain reaction. (DOCX) [file pone.0193173.s002.docx]

S2 Table: Thermal cycler conditions reverse transcription, preamplification and qPCR.

| 1. Reverse transcription | | | 2. Preamplification | | | 3. qPCR | | |
| --- | --- | --- | --- | --- | --- | --- | --- | --- |
| Stage | Temperature | Time | Stage | Temperature | Time | Stage | Temperature | Time |
| Cycle (40 cycles) | 16°C | 2:00 | Hold | 95°C | 10:00 | Hold | 50°C | 2:00 |
|  | 42°C | 1:00 | Hold | 55°C | 2:00 | Hold | 94.5°C | 10:00 |
|  | 50°C | 0:01 | Hold | 72°C | 2:00 | Cycle (40 cycles) | 97°C | 0:30 |
| Hold | 85°C | 5:00 | Cycle (12 cycles) | 95°C | 0:15 |  | 59.7°C | 01:00 |
|  |  |  |  | 60°C | 4:00 |  |  |  |
|  |  |  | Hold | 99.9°C | 10:00 |  |  |  |
